# Remote ischaemic conditioning and early changes in plasma creatinine as markers of one year kidney graft function—A follow-up of the CONTEXT study

**DOI:** 10.1371/journal.pone.0226882

**Published:** 2019-12-30

**Authors:** Marie B. Nielsen, Nicoline V. Krogstrup, Mihai Oltean, Gertrude J. Nieuwenhuijs-Moeke, Frank J. M. F. Dor, Henrik Birn, Bente Jespersen

**Affiliations:** 1 Department of Renal Medicine, Aarhus University Hospital, Aarhus, Denmark; 2 Departments of Clinical Medicine, Aarhus University, Aarhus, Denmark; 3 Department of Renal Medicine, Herlev Hospital, Herlev, Denmark; 4 The Transplant Institute, Sahlgrenska University Hospital, Gothenburg, Sweden; 5 Department of Anaesthesiology, University of Groningen, University Medical Center Groningen, Groningen, The Netherlands; 6 Division of HPB & Transplant Surgery, Department of Surgery, Erasmus MC, University Medical Center, Rotterdam, The Netherlands; 7 Imperial College Renal and Transplant Centre, Hammersmith Hospital, Imperial College, London, United Kingdom; 8 Department of Biomedicine, Aarhus University, Aarhus, Denmark; Universita degli Studi di Perugia, ITALY

## Abstract

**Background:**

Ischaemia-reperfusion injury in kidney transplantation leads to delayed graft function (DGF), which is associated with reduced long term graft function. Remote ischaemic conditioning (RIC) improved early kidney graft function in a porcine model of donation after brain death and was associated with improved long-term cardiac outcome after myocardial ischaemia. This randomised, double-blinded trial evaluated the effect of RIC on kidney graft outcome in the first year, and examined the predictive value of a new measure of initial kidney graft function, i.e. the estimated time to a 50% reduction in plasma creatinine post-transplantation (tCr50).

**Methods:**

A total of 225 patients undergoing deceased donor kidney transplantation were randomised to RIC or a sham procedure performed prior to kidney reperfusion. Up to four repetitive cycles of five minutes of leg ischaemia and five minutes of reperfusion were given. GFR, plasma creatinine, cystatin C and neutrophil gelatinase associated lipocalin (NGAL) were measured at three and twelve months and estimated GFR was calculated using four different equations. Other secondary outcomes were identified from patient files.

**Results:**

RIC did not affect GFR or other outcomes when compared to the sham procedure at three or twelve months. tCr50 correlated with one year graft function (p<0.0001 for both mGFR and eGFR estimates). In contrast, DGF i.e. “need of dialysis the first week” did not correlate significantly with one year GFR.

**Conclusion:**

RIC during deceased donor kidney transplantation did not improve one year outcome. However, tCr50 may be a relevant marker for studies aiming to improve graft onset.

**Trial registration:**

www.ClinicalTrials.gov Identifier: NCT01395719.

## Introduction

Delayed graft function (DGF) is a consequence of ischaemia-reperfusion injury and a common complication after deceased donor kidney transplantation [1,2]. It is associated with an increased incidence of post-transplant complications, acute rejection, reduced long-term graft outcome and graft loss [3–5].

Remote ischaemic conditioning (RIC) protects against ischaemia-reperfusion injury in various tissues in both experimental and some clinical studies, particularly in the heart [6,7], but also in the transplanted kidney in our porcine model [8]. It is believed that RIC sends a protective signal to a remote organ or tissue that is transferred via humoral, neuronal or other systemic pathway to the cells in the target organ. RIC applied during myocardial infarction has been associated with a better long-term outcome when evaluated at a mean of 3.8 years follow-up [9]. Indeed, in our porcine donation after brain death transplantation model, RIC improved early glomerular filtration rate (GFR) and renal plasma perfusion [8].

The multi-centre, randomised, controlled clinical study CONTEXT [10] evaluated the effect of recipient RIC in deceased donor kidney transplantation. Many definitions of delayed graft function have been proposed based on reduction of creatinine level or need for dialysis [11]. As previously published, RIC had no effect on early kidney graft function measured as the estimated time to a 50% reduction in plasma (P-) creatinine (tCr50) [12] or the incidence of DGF defined as the need for dialyses within the first week after transplantation [13]. In this follow up of the CONTEXT study, we evaluated the effect of RIC on kidney graft function, rejection rate, new onset diabetes after transplantation (NODAT), patient survival, and the kidney injury marker neutrophil gelatinase associated lipocalin (NGAL) [14,15] at three and twelve months post-transplantation. We further examined whether tCr50 predicted GFR at twelve months. GFR was measured and estimated based on P-creatinine and P-cystatin C.

## Materials and methods

### Study design

The study design of CONTEXT (www.ClinicalTrials.gov Identifier: NCT01395719) was previously described in detail [10]. A total of 225 adult patients undergoing deceased donor kidney transplantation were included at four centres: Aarhus, Denmark; Gothenburg, Sweden; and Groningen and Rotterdam in the Netherlands [13]. All patients provided written informed consent. The study was approved by the National Committee on Health Research Ethics (Denmark), Regional Ethical Board (Sweden) and METCUMCG (the Netherlands).

Inclusion criteria were deceased donor kidney transplantation and age ≥ 18 years. Exclusion criteria were double kidney transplant, risk of lower limb ischaemia during RIC, and AV-fistula in the leg where RIC was performed.

Patients were randomised to receive either RIC or sham-RIC during surgery. The RIC procedure consisted of up to four cycles of inflation and deflation of a tourniquet around the thigh contralateral to the transplantation side. This resulted in cycles of five minutes ischaemia and five minutes reperfusion performed just prior to reperfusion of the kidney. Kidney graft function was evaluated at three and twelve months post-transplantation. Additional information on patient and graft survival, the need for dialysis, rejection episodes and NODAT was obtained from patient records.

### Blood and urine

EDTA-blood and urine samples were collected, centrifuged at 2800G at 4°C for ten minutes and stored at -80°C until further analysed. P-NGAL was measured using a particle-enhanced turbidimetric immunoassay (^®^BioPorto A/S, Hellerup, Denmark). P-cystatin C was measured using a turbidimetric immunoassay (Siemens Healthcare A/S, Ballerup, Denmark). P-creatinine, U-creatinine and U-albumin were measured using automated, standard clinical assays at the Department of Clinical Biochemistry, Aarhus University Hospital. tCr50, which was the primary end point of the CONTEXT study, was calculated from post-transplant changes in P-creatinine as previously described [13] and DGF was defined as the need for dialysis within the first week after transplantation.

### Glomerular filtration rate

Measured GFR (mGFR) was determined using ^51^chrome-ethylenediamine tetraacetic acid [16] (^51^Cr-EDTA) or iodothalamate plasma clearance. Estimated GFR (eGFR) was calculated using the following equations without correction for race (>90% of the patients were Caucasian): a) the simplified MDRD creatinine based formula [17]; b) the CKD-EPI creatinine based formula (eGFRCr) [18]; c) the CKD-EPI cystatin C based formula (eGFRCys) [19]; and d) the CKD-EPI creatinine and cystatin C based formula (eGFRCr-Cys) [19].

### Statistical analyses

The mGFR, eGFR estimates, and P-NGAL at three and twelve months were analysed using repeated measurement analysis of variance or linear regression. A linear mixed regression effects model was used to compare log transformed outcomes between the RIC and sham-RIC groups, with RIC and centre as fixed effects, and donor as random effect. Data are presented as n (%), mean (SD), median (IQR) or estimated median (95% CI). The Chi-square test or Fisher’s exact test was used to examine the significance of categorical outcomes. Continuous variables were correlated using simple linear regression. Data were analysed in Stata/IC 12.1 for Mac (Copyright 1985–2011 Stata-Corp LP, College Station, TX).

## Results

### Inclusion

**Fig 1:**
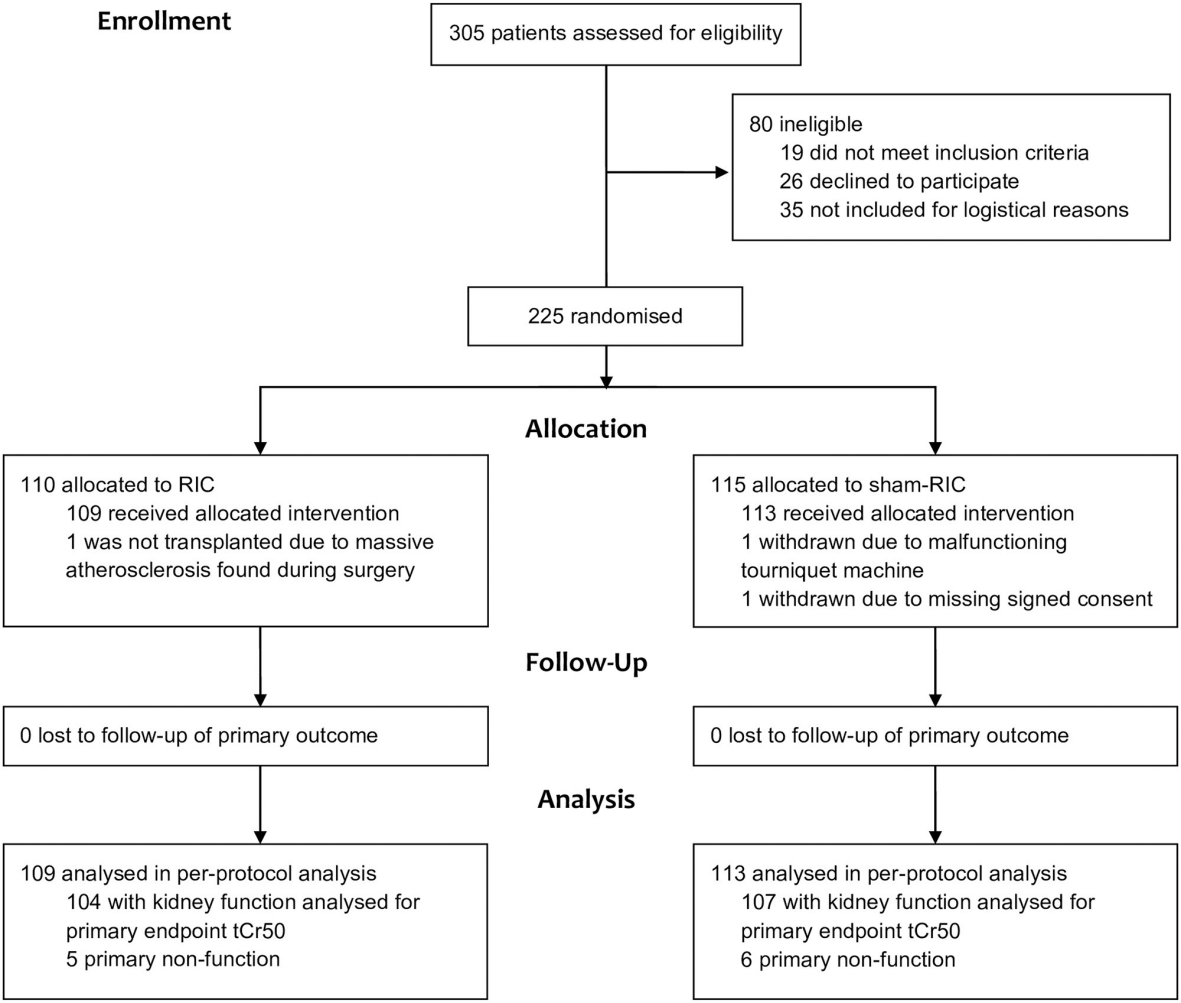
CONSORT flow diagram of inclusion and randomisation.

225 patients were included from June 12, 2011, to December 28, 2014. Three patients were withdrawn from the study during the transplant surgery, leaving a total of 222 patients for the per-protocol analysis (Fig 1).

### RIC did not affect GFR or other outcomes at three months and one year

As previously reported, baseline data, tCr50 and the incidence of DGF were similar in the RIC and sham-RIC groups (Table 1) [13].

**Table 1:** Recipient and donor characteristics. Baseline characteristics, tCr50 and the incidence of DGF. Data are mean (standard deviation), n (%), or median (interquartile range). No statistically significant differences were identified between the groups. tCr50 = estimated time to a 50% reduction in plasma creatinine post-transplantation. DGF = delayed graft function defined as need of dialysis first week post-transplantation. DBD = donation after brain death. DCD = donation after circulatory death. Remuzzi score on biopsies taken 30 min after reperfusion of the graft (baseline-biopsy). + score if including only biopsies with minimum six or ten glomeruli in the analysis.

Recipient	RIC (n = 109)	Sham-RIC (n = 113)
Gender, male	65 (60%)	69 (61%)
Age (years)	58.1 (49.5–65.0)	61.4 (49.4–66.6)
Preemptive transplantation	17 (16%)	23 (20%)
First transplantation	95 (87%)	98 (87%)
Diabetes mellitus	13 (12%)	13 (12%)
Total HLA-A, B, DR mismatches		
0	6 (5.5%)	6 (5.3%)
1–2	17 (16%)	24 (21%)
3–4	70 (64%)	58 (51%)
5–6	16 (15%)	25 (22%)
Immunosuppression at discharge		
Tacrolimus	98 (90%)	106 (94%)
Mycophenolate mofetil	105 (96%)	111 (98%)
Corticosteroids	101 (93%)	109 (96%)
Cyclosporine	7 (6.4%)	5 (4.4%)
None (graft loss)	4 (3.7%)	2 (1.8%)
**Donor**		
Gender, male	60 (55%)	61 (54%)
Age (years)	58 (52–66)	58 (52–65)
DBD	98 (90%)	102 (90%)
DCD	11 (10%)	11 (10%)
Cause of death, DBD (n = 98)		
Cerebrovascular insult	64 (65%)	70 (69%)
Cerebral anoxia	22 (22%)	20 (20%)
Trauma	11 (11%)	12 (12%)
Benign cerebral neoplasm	1 (1.0%)	0
Warm ischemia time DCD donor (min)	20 (15–21)	14 (13–18)
Missing data	0	1
Cold ischemia time (hours), DBD+DCD	13.3 (4.0)	13.6 (4.8)
n > 24h	3 (2.8%)	4 (3.5)
Missing data	2 (1.9%)	1 (0.9%)
Remuzzi score (baseline-biopsy)		
Score all biopsies (n = 91&94)	2 (1–3)	2 (1–4)
Score 6 glom+ (n = 80&82)	2 (1–3.5)	2 (1–4)
Score 10 glom+ (n = 53&63)	3 (1–4)	3 (1–4)
Missing data^a^	18 (17%)	19 (17%)
**Post-transplantation**		
tCr50 (hours) (n = 104&107)	122 (98–151)	112 (91–139)
DGF	36 (33%)	40 (35%)

^a^Biopsies were either not performed (n = 22) or insufficient (n = 15).

This extended analysis showed that both the three month and one-year graft function were also similar, with no significant differences either in mGFR or eGFR between RIC and sham-RIC (Table 2). Also, no differences between groups were observed with respect to mortality, need for dialysis, rejections, NODAT or the renal injury biomarker P-NGAL. Analyses including only the youngest 50% of the transplant recipients [20] also did not reveal any tendencies on effects of RIC regarding tCr50 (RIC: mean 129 hrs, 95% confidence interval (CI) 96–172, n = 60; vs. sham-RIC: 92 hrs, 95% CI 63–135, n = 47; p = 0.16) or mGFR at one year (RIC: 56 ml/min/1.73m^2^ 95% CI 49–63, n = 40; vs. sham-RIC: 52 ml/min/1.73m^2^, 95% CI 46–59, n = 32; p = 0.43).

**Table 2:** Outcomes at three and twelve months. Recipient and renal graft outcomes at three and twelve months depending on RIC or sham procedure. Values are n (%), estimated median (95% CI) or mean (95% CI). mGFR and eGFR values are ml/min/1.73m^2^. mGFR, measured GFR; eGFR, estimated GFR.

Three months	RIC (n = 109)		Sham-RIC (n = 113)		p
Deaths	1 (0.9%)		0		0.49
On dialysis	7/109 (6.4%)		7/113 (6.2%)		0.94
Rejection^a^	10 (9.2%)		9 (8.0%)		0.74
NODAT^b^	8 (7.3%)		12 (10.1%)		0.39
P-creatinine, μmol/L	136 (127–146)	n = 101	140 (131–149)	n = 106	0.60
P-NGAL, μg/L	163 (147–180)	n = 83	176 (160–192)	n = 90	0.25
P-cystatin C, mg/L	2.00 (1.88–2.13)	n = 83	2.17 (2.01–2.34)	n = 90	0.11
mGFR	41 (37–44)	n = 78	43 (39–47)	n = 70	0.35
eGFR, MDRD^c^	42 (39–46)	n = 101	40 (38–44)	n = 106	0.34
eGFRCr^d^	45 (41–48)	n = 101	42 (39–46)	n = 106	0.30
eGFRCys^e^	30 (28–33)	n = 83	27 (25–30)	n = 90	0.09
eGFRCr-Cys^f^	36 (33–39)	n = 83	33 (31–36)	n = 90	0.21
**Twelve months**					
Deaths	2 (1.8%)		2 (1.8%)		1.00
On dialysis	8/109 (7.3%)		10/113 (8.8%)		0.68
Rejection^a^	17 (15.6%)		14 (12.4%)		0.49
NODAT^b^	11 (10.1%)		14 (12.4%)		0.59
P-creatinine, μmol/L	135 (125–145)	n = 99	134 (124–144)	n = 101	0.89
P-NGAL, μg/L	179 (163–197)	n = 79	181 (166–197)	n = 79	0.87
P-cystatin C, mg/L	1.85 (1.72–1.99)	n = 79	1.92 (1.78–2.07)	n = 78	0.48
mGFR	46 (41–51)	n = 67	44 (39–48)	n = 74	0.44
eGFR, MDRD^c^	43 (39–46)	n = 99	42 (39–45)	n = 101	0.77
eGFRCr^d^	45 (41–49)	n = 99	44 (40–48)	n = 101	0.75
eGFRCys^e^	34 (31–37)	n = 80	33 (30–36)	n = 77	0.56
eGFRCr-Cys^f^	38 (35–42)	n = 80	38 (34–41)	n = 77	0.85
U-albumin/creatinine, mg/g	52 (37–73	n = 68	46 (32–66)	n = 68	0.63

^a^Total number of patients with one or more rejections at three and twelve months.

^b^NODAT: total number of patients with NODAT (new-onset diabetes after transplantation) at three and twelve months.

^c^The simplified MDRD creatinine based formula.

^d^The CKD-EPI creatinine based formula (eGFRCr).

^e^The CKD-EPI cystatin C based formula (eGFRCys).

^f^The CKD-EPI creatinine and cystatin C based formula (eGFRCr-Cys).

### tCr50 as predictor of GFR at twelve months

The tCr50, which is a previously developed marker of early graft function for the CONTEXT study [13], correlated weakly, but highly significantly, with both mGFR and eGFR at twelve months (Table 3), i.e. a longer tCr50 is associated with a lower GFR at one year. In contrast, the presence or absence of DGF, i.e. need of dialysis first week post-transplant, did not correlate significantly with mGFR (DGF: 48 ml/min/1.73m^2^ 95% CI 44–52, n = 106; vs. no DGF 52 ml/min/1.73m^2^ 95% CI 45–58, n = 35; p = 0.39) at twelve months.

**Table 3:** tCr50 and kidney graft function at twelve months. Linear regression identifying a negative correlation between tCr50 and kidney graft function at twelve months. tCr50 = the estimated time to a 50% reduction in P-creatinine. mGFR = measured GFR.

	tCr50			
	n	p	r	r^2^_adj._
mGFR	141	<0.0001	-0.39	0.15
eGFR, MDRD^a^	200	<0.0001	-0.30	0.09
eGFRCr^b^	200	<0.0001	-0.30	0.09
eGFRCys^c^	157	<0.0001	-0.34	0.11
eGFRCr-Cys^d^	157	<0.0001	-0.36	0.13

^a^The simplified MDRD creatinine based formula.

^b^The CKD-EPI creatinine based formula (eGFRCr).

^c^The CKD-EPI cystatin C based formula (eGFRCys).

^d^The CKD-EPI creatinine and cystatin C based formula (eGFRCr-Cys).

## Discussion

In this randomised clinical CONTEXT trial, RIC did not improve GFR at one year after renal transplantation. Also, RIC did not affect the risks of graft loss, death, rejection or NODAT. This suggests that RIC does not offer any long-term protection in deceased donor kidney transplantation. At the same time tCr50, the primary endpoint of the CONTEXT study, was shown to correlate significantly with GFR at one year.

To our knowledge, the CONTEXT study is the largest investigation to date for RIC in deceased donor kidney transplantation using a multicentre approach. The results were further strengthened by the randomised double blinded design as well as the use of both mGFR and P-cystatin C which are considered to provide more accurate estimates of GFR compared to eGFR based on P-creatinine alone.

Transplantation from deceased donors is accompanied by more profound ischaemia reperfusion injury and an increased risk of DGF compared to living donor kidney transplantation. Still, our results are clear and in concordance with findings in living donor transplantation reported in the REPAIR study [21], which investigated the effect of early and late phase RIC applied to both the donor and recipient at different time points prior to transplantation. The REPAIR study also failed to demonstrate any significant effect of RIC on kidney function at one year. However, a trend towards higher GFR (mGFR 58.3 vs. 55.9 mL/min/1.73m2, p = 0.13) was observed in the group receiving RIC with a similar timing to the one used in the CONTEXT study, when compared to the control group. Other and smaller studies have also not been able to demonstrate a beneficial effect of RIC in renal transplantation either on patient or kidney graft outcomes nor on various renal biomarkers [22–25].

Thus, the positive short term effects of RIC reported by our group in a porcine donation after brain death transplant model [8] were not reproduced in patients in respect of either the early graft function [10] or GFR at one year. Although animal studies have resulted in promising results to ameliorate renal ischaemia reperfusion injury, more research is required to understand the mechanisms involved as little of the positive animal research data can be replicated in the clinic nor can it be shown to improve transplant outcome [26]. A number of factors, such as uraemia, co-morbidity including diabetes, age [20], anaesthesia [27] or other yet unidentified factors might explain this lack of effect in the clinical setting and they may also blur potential beneficial effects of RIC in patients. Most animal studies are performed in healthy animals not receiving immunosuppressants or other drugs that may potentially influence outcomes. It is possible that such medications may either block the protective mechanism induced by the RIC or could have yielded other anti-ischaemic effects [28–30], thus blunting or obliterating any beneficial effect of RIC. In addition to recipient-related and potentially confounding factors, there are additional donor-related variables that may influence and attenuate the effect of RIC such as organ injury and inflammatory activation during brain death [31]. In most animal models organs are retrieved shortly after brain death, e.g. after four hours in our porcine model [8], while in humans retrieval is often delayed. The protective mechanisms induced by RIC (if any) were unfortunately insufficient to result in meaningful clinical effects.

It is possible that another explanation as to why our study failed to show a protective effect of RIC may be the timing of application of the RIC stimulus which could be important for the protective effects. Transplantation is a unique setting for the simple reason that the organ is not connected to the recipient until reperfusion. It is believed that RIC may provide a first window of protection conferred by humoral factors released from the conditioned tissue. It is possible that such factors were no longer in circulation at the time of reperfusion although this was only 38 (IQR 19–54) minutes after the end of the RIC procedures last cuff release [13]. The suggested humoral factors have short plasma half-lives [32]. Also, since the transplanted kidney is denervated, any neuronal pathway for RIC effects is excluded.

In addition our study demonstrated a weak, but highly statistically significant, correlation between tCr50 and both mGFR and eGFR at twelve months. No such correlation was demonstrated when comparing the incidence of DGF with GFR at twelve months. This may suggest that tCr50 is a more sensitive marker of the impact of early graft function on long term GFR than the commonly used definition of DGF, i.e. the need for dialysis within the first week post-transplantation. Thus, tCr50 although not simple to calculate [13] could be of use in future studies on interventions to improve early and long term graft function. In addition to tCr50’s correlation with one-year GFR, it has the advantage of being a continuous variable allowing all patients to be included in the analysis except for the few who never obtain a 50% reduction in P-creatinine.

In conclusion, our results indicate that RIC of the organ recipient does not offer any advantages in kidney transplantation either on short or one year outcomes. tCr50 is negatively associated with GFR at one year and may be used as a marker of early graft function with an impact on long term outcome.

## Supporting information

S1 Checklist Consort 2010 checklist of information to include when reporting a randomised trial*. (DOC)

## Abbreviations

^51^Cr-EDTA: ^51^chrome-ethylenediamine tetraacetic acid
DBD: donation after brain death
DCD: donation after circulatory death
DGF: delayed graft function
eGFR: estimated glomerular filtration rate
eGFR: glomerular filtration rate
eGFRCr: the CKD-EPI creatinine based GFR formula
eGFRCr-CYS: the CKD-EPI creatinine and cystatin C based GFR formula
eGFRCys: the CKD-EPI cystatin C based GFR formula
mGFR: measured glomerular filtration rate
NGAL: neutrophil gelatinase associated lipocalin
NODAT: new onset diabetes mellitus after transplantation
P-: plasma
RIC: remote ischaemic conditioning
tCr50: the estimated time to a 50% reduction in plasma creatinine
